# Reduced IgM natural autoantibody levels against heat shock proteins are associated with disease severity in juvenile idiopathic arthritis

**DOI:** 10.3389/fimmu.2026.1845910

**Published:** 2026-07-17

**Authors:** Bernadett Mosdósi, Vivien Mátis, Ádám Győri, Panna Peti, Szabina Erdő-Bonyár, Timea Berki, Diana Simon

**Affiliations:** 1 Department of Pediatrics, Medical School, University of Pecs, Pecs, Hungary; 2 Department of Immunology and Biotechnology, Medical School, University of Pecs, Pecs, Hungary

**Keywords:** autoimmunity, heat shock protein 60, heat shock protein 70, juvenile idiopathic arthritis, natural autoantibodies

## Abstract

**Introduction:**

Natural autoantibodies (nAAbs) contribute to immune homeostasis and may be altered in patients with autoimmune diseases. While anti-heat shock protein (Hsp) antibodies have been previously described in juvenile idiopathic arthritis (JIA), their relationship with disease severity and treatment effects during remission remains unclear. This study aimed to measure the serum levels of IgM and IgG nAbs against heat shock protein (Hsp) 60 and Hsp70 in children with JIA in remission (mean age: 7.41 years, range:1.27-14.54 years) and to explore their associations with disease subtype, severity, and treatment.

**Methods:**

Serum samples from 47 children with JIA in remission and 22 apparently healthy controls (HC) were analyzed. Patient groups were stratified according to the treatment regimen (methotrexate monotherapy versus methotrexate plus adalimumab) and disease subtype and severity. Anti-Hsp60 and anti-Hsp70 IgM and IgG antibodies were measured u**s**ing an in-house indirect ELISA.

**Results:**

No significant differences in anti-Hsp60 or anti-Hsp70 nAAb levels were observed between patients with JIA and apparently healthy controls or between treatment groups. However, severity-based analyses revealed significantly lower anti-Hsp60 and anti-Hsp70 IgM levels in patients with polyarticular JIA than in those with milder oligoarticular forms and in apparently healthy controls. No significant differences were observed in the anti-Hsp60 or anti-Hsp70 IgG levels.

**Conclusion:**

Reduced levels of natural IgM autoantibodies against Hsp60 and Hsp70 are associated with more severe JIA phenotypes during remission, supporting the potential immunoregulatory and protective roles of natural IgM autoantibodies in JIA.

## 1 Introduction

Juvenile idiopathic arthritis (JIA) is the most common chronic inflammatory rheumatic disease in children. JIA is defined as joint inflammation of unknown origin that begins before the age of 16 years and persists for at least 6 weeks (1). According to the International League of Associations for Rheumatology (ILAR) classification, JIA includes several clinical subtypes, such as oligoarticular, polyarticular, enthesitis-related, psoriatic arthritis, and systemic JIA, which differ in clinical course, severity, epidemiological characteristics, and immunopathological features (2). The epidemiology of JIA varies significantly across geographic regions, ethnic groups, and classification systems. The global incidence is approximately 1.6–23 cases per 100,000 children per year, while prevalence estimates range from 3.8–400 cases per 100,000 children (3). In Western countries, oligoarticular JIA is the most common subtype, whereas in certain Asian, African, and Latin American populations, polyarticular and enthesitis-related forms are more common (4).

The severity of juvenile idiopathic arthritis ranges from relatively mild, oligoarticular forms with a favorable prognosis to severe systemic or polyarticular disease characterized by severe chronic inflammation and joint damage (5). Current treatment strategies include nonsteroidal anti-inflammatory drugs, conventional disease-modifying antirheumatic drugs, such as methotrexate and sulfasalazine, and biologics (4, 6). Advances in early aggressive therapy have significantly improved the long-term outcomes of children with JIA. The long-term outcomes of JIA range from sustained remission to persistently active disease associated with joint damage, disability, impaired quality of life, and extra-articular complications in adulthood (5). Although the precise mechanisms driving disease initiation and perpetuation remain incompletely understood, increasing evidence implicates immune dysregulation and altered self-recognition in the pathogenesis of JIA (7). While pathological autoantibodies have been extensively studied in JIA, the potential role of natural autoantibodies (nAAbs) remains unclear (8–10).

The nAAb repertoire is genetically regulated and capable of recognizing self- or pathogen-derived antigens that have been conserved throughout evolution (11). nAAbs typically recognize structures such as heat shock proteins, cytoskeletal proteins, metabolic enzymes, and nuclear components (12, 13). Natural autoantibodies are primarily produced by B1 and marginal zone B cells and are characterized by polyreactivity and low affinity (11, 13). nAAbs are detectable in healthy individuals and are considered important components of physiological immune homeostasis (12, 14, 15). Nevertheless, quantitative and qualitative alterations in their repertoire, specificity, and functional relevance have been reported in several autoimmune diseases (10, 16, 17). In our previous study, we found that the levels of IgM nAAbs were elevated in anti-dsDNA IgM-positive patients with systemic lupus erythematosus, suggesting that these immunoglobulins may play a protective role (18). We also described that the level of IgG nAbs was increased in systemic sclerosis in patients with active disease (19).

Among the emerging contributors, heat shock proteins (Hsps) have garnered attention for their dual role as intracellular chaperones and immunomodulatory molecules capable of bridging innate and adaptive immunity (20). Zlacka et al. previously described elevated levels of anti-Hsp70 IgG in rheumatoid factor positive JIA patients. Anti-Hsp70 IgG levels were shown to correlate with disease severity (8). Understanding whether and how disease-modifying interventions influence anti-HSP nAAb levels could help identify novel biomarkers of treatment response and provide further insights into the interaction between therapeutic immunomodulation and autoimmunity. Notably, while conventional therapies such as methotrexate and biologics targeting tumor necrosis factor alpha (TNFα) have revolutionized JIA management, their impact on nAAb repertoires, including anti-Hsp autoantibodies, remains unknown. Our study aimed to explore the potential relationships between anti-Hsp60 and anti-Hsp70 nAAb levels and treatment regimens in JIA patients in remission (mean age: 7.41 years, range:1.27-14.54 years), considering disease phenotypes.

## 2 Materials and methods

### 2.1 Study design and participants

**Figure 1:**
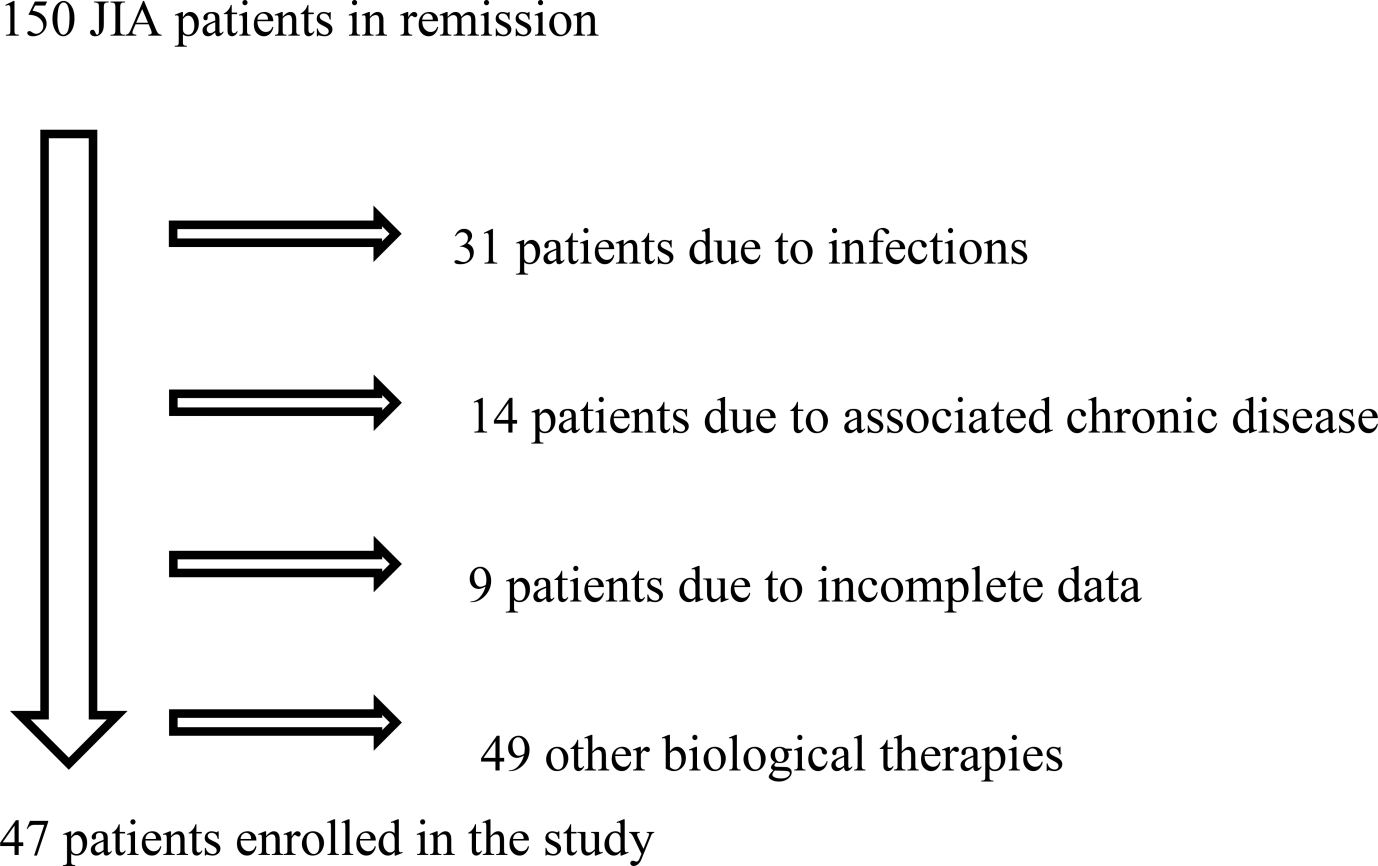
Participant selection flow diagram.

This retrospective study was conducted at the University of Pécs (PTE), Department of Pediatrics, and the Institute of Immunology and Biotechnology. The study included children diagnosed with JIA who were clinically in remission at the time of blood sampling. Clinical remission was defined based on the absence of active arthritis, absence of morning stiffness, and normal routine inflammatory laboratory parameters (C-reactive protein, erytrocyte sedimentation rate and white blood cell count), in accordance with established clinical criteria in accordance with the Wallace criteria for inactive disease in JIA (20). The exclusion criteria were an infectious disease present during the study period or within the preceding two months, other autoimmune or chronic systemic diseases, incomplete clinical or laboratory data, and receiving biological therapies other than adalimumab. Initially, 150 patients in remission were enrolled in the study; however, 31 patients were excluded due to infectious diseases, 14 due to associated chronic diseases, 9 due to incomplete data, and 49 due to other biological therapies (**Figure 1**).

The 47 patients participating in the study were classified into four main clinical subtypes: oligoarticular (OA), polyarticular (PA), enthesitis-associated arthritis (ER)), and systemic-onset JIA. Additionally, two subsets were defined in the OA group: persistent OA (pOA) and extended OA (eOA). Twenty-two apparently healthy controls (HC) were recruited from children attending routine health examinations or elective visits, with no history of autoimmune, inflammatory, or chronic disease. Patients were further stratified according to the treatment regimen into the MTX monotherapy (n=19) and MTX and adalimumab combination therapy (MTX/ADA) (n=28) groups. The demographic data, subtype classification, disease duration, and duration of therapy are summarized in **Table 1**.

**Table 1:** Demographic data and subset classification of patients and apparently healthy controls.

Demographic data	MTX monotherapy (n=19)	MTX/ADA combination therapy (n=28)	Apparently HC (n=22)
Average age (year)	7.12 (2.31- 11.93)	7.47 (3.1-11.84)	12.40 (8.38-16.42)
Male, n (%)	5 (26.3)	12 (46.15)	13 (59.09)
Female, n (%)	14 (73.68)	16 (57.14)	9 (40.9)
Duration of the JIA (year)	2.00 (0.25-13.83)	3.80 (0.91-11)	–
Duration of therapy (year)	2.00 (0.25-13.83)	1.88 (0.25-7.58)	–
Subtype of JIA			
Oligoarticular (OA), n (%) • persistent OA (pOA) • extended OA (eOA) Polyarticular (PA), n (%) Enthesitis-related arthritis (ERA), n (%) Systemic onset JIA, n (%)	13 (68.4) 5 8 2 (10.5) 1 (5.3) 3 (15.8)	14 (50) 5 9 8 (28.6) 6 (21.4) -	- - - - - -

JIA, juvenile idiopathic arthritis; ERA, Enthesitis-associated arthritis; HC, healthy control, MTX, methotrexate; MTX/ADA, methotrexate + adalimumab; n, number.

### 2.2 Laboratory tests

Routine laboratory parameters (blood count, sedimentation rate, C-reactive protein, liver and kidney function, blood lipids, and blood sugar levels) and serum immunoglobulin levels were measured (**Table 2**). None of the test results indicated inflammation in any patient.

**Table 2:** Routine laboratory parameters.

Laboratory parameters (reference range)	MTX monotherapy (n=19) Mean+/-SD, (min;max)	MTX/ADA combination therapy (n=28) Mean+/-SD, (min;max)	HC (n=22)
Sedimentation rate (10 mm/hour)	5.31 ± 2.18 (2;9)	4.76 ± 2.19 (2;9)	4.77 ± 2.17 (2;9)
C-reactive protein (5 mg/l >)	1.52 ± 2.20 (0.1;4.3)	1.15 ± 1.53 (0.1;3.4)	0.75 ± 0.74 (0.1;1.8)
White blood cell count (4.5-11.0 G/l)	7.89 ± 3.10 (5.39;10,3)	6.78 ± 1.56 (4.51;9.96)	7.06 ± 1.55 (4.67;9.11)
Neutrophil granulocyte (1.5-8.0 G/l)	3.93 ± 1.57 (1.87;6.55)	3.47 ± 1.35 (1.57;7.73)	3.79 ± 1.27 (1.52;6.25)
Lymphocyte (1.25-8.0 G/l)	2.89 ± 1.52 (1.33;7.75)	2.65 ± 0.90 (1.42;4.88)	2.61 ± 0.83 (1.47;4.67)
Hemoglobin (120–145 g/l)	129.72 ± 9.16 (121;137)	132.04 ± 8.26 (120;144)	134.55 ± 7.67 (120;145)
Hematocrit (Hct) (35-47%)	38.30 ± 2.89 (36.2;45.6)	39.68 ± 2.80 (6.1;46.6)	40.18 ± 3.19 (36.4;46.3)
IgM antibody (0.8-1.9 g/l)	1.31 ± 0.45 (0.84;1.85)	1.18 ± 0.38 (0.8;1.76)	1.04 ± 0.36 (0.8;1.8)
IgG antibody (8.0-12.0 g/l)	9.88 ± 1.56 (8.11;11.5)	11.49 ± 1.28 (8.25,12.0)	10.17 ± 1.78 (8.09;12.0)
IgA antibody (1.0-2.0 g/l)	1.45 ± 0.56 (1.1;2.0)	1.59 ± 0.40 (1.08;2.0)	1.47 ± 0.37 (1.02;2.0)

HC, healthy control; Ig, immunoglobulin; MTX, methotrexate; MTX/ADA, methotrexate + adalimumab; SD, standard deviation.

### 2.3 Detection of anti- Hsp60 and anti-Hsp70 natural autoantibodies

Serum IgM and IgG autoantibodies against Hsp60 and Hsp70 were measured using an in-house indirect enzyme-linked immunosorbent assay (ELISA) according to a previously described protocol (21).

Briefly, high-binding 96-well plates (Nunc Maxisorp) were coated with human Hsp60 (Abcam, Waltham, Boston, MA, USA) or human Hsp70 (Abcam, Waltham, Boston, USA) at a concentration of 1 μg/mL in ELISA Coating Buffer (Bio-Rad, Hercules, CA, USA) at 4 °C overnight. The wells were blocked using an alternative combined blocking buffer (0.5% polyvinyl alcohol solution combined with bovine gelatin solution at a ratio of 2:1) at room temperature (RT) for 2 h. Following washing with PBS + 0.05% Tween WB), 1:200 pre-diluted sera were incubated for 1 h at 37 °C. Secondary antibodies, horseradish peroxidase-conjugated polyclonal rabbit anti-human IgG and IgM antibodies (Agilent-Dako, Santa Clara, CA, USA), were incubated at 37 °C for 40 min. The 3,3′,5,5′-tetramethylbenzidine (TMB) substrate solution (Sigma-Merck, Munich, Germany) was used to develop the reaction, which was stopped using 1 M H_2_SO_4_. Optical densities (OD) were measured at λ = 450/620nm using the BEP2000 Advanced automated system (Siemens, Marburg, Germany). Background OD values, determined from control wells reflecting assay background and non-specific binding, were subtracted from the OD values obtained for patients’ sera before further analysis. Negative controls were included to assess baseline reactivity, and commercially available anti-Hsp antibodies and high-reactivity serum samples were used as positive controls. Due to the absence of internationally standardized reference materials for nAAb quantification, titers were expressed in arbitrary units (Units) relative to in-house internal standards calibrated against stock preparations.

### 2.4 Statistical analysis

Statistical analyses were performed using the Statistical Package for the Social Sciences (SPSS) version 27.0 statistics package (IBM, Armonk, NY, USA) program. The data distribution was assessed for normality using the Shapiro–Wilk test because of the small sample size. The sample size was determined based on the availability of eligible participants. As the data were not normally distributed, non-parametric tests were used. The Mann–Whitney U test was used for comparisons between two groups while the Kruskal–Wallis test was used for comparisons among more than two groups followed by pairwise comparisons using Dunn’s test with Bonferroni correction. Statistical significance was set at p < 0.05.

### 2.5 Ethics

The study was conducted in accordance with the Declaration of Helsinki and approved by the Regional Clinical Research Ethics Committee, University of Pecs (protocol number:9603-PTE2023, approval date:19.05.2023). Due to participants being minors, written informed consent was obtained from the legal guardians of all enrolled participants, including apparently healthy controls.

## 3 Results

**Figure 2:**
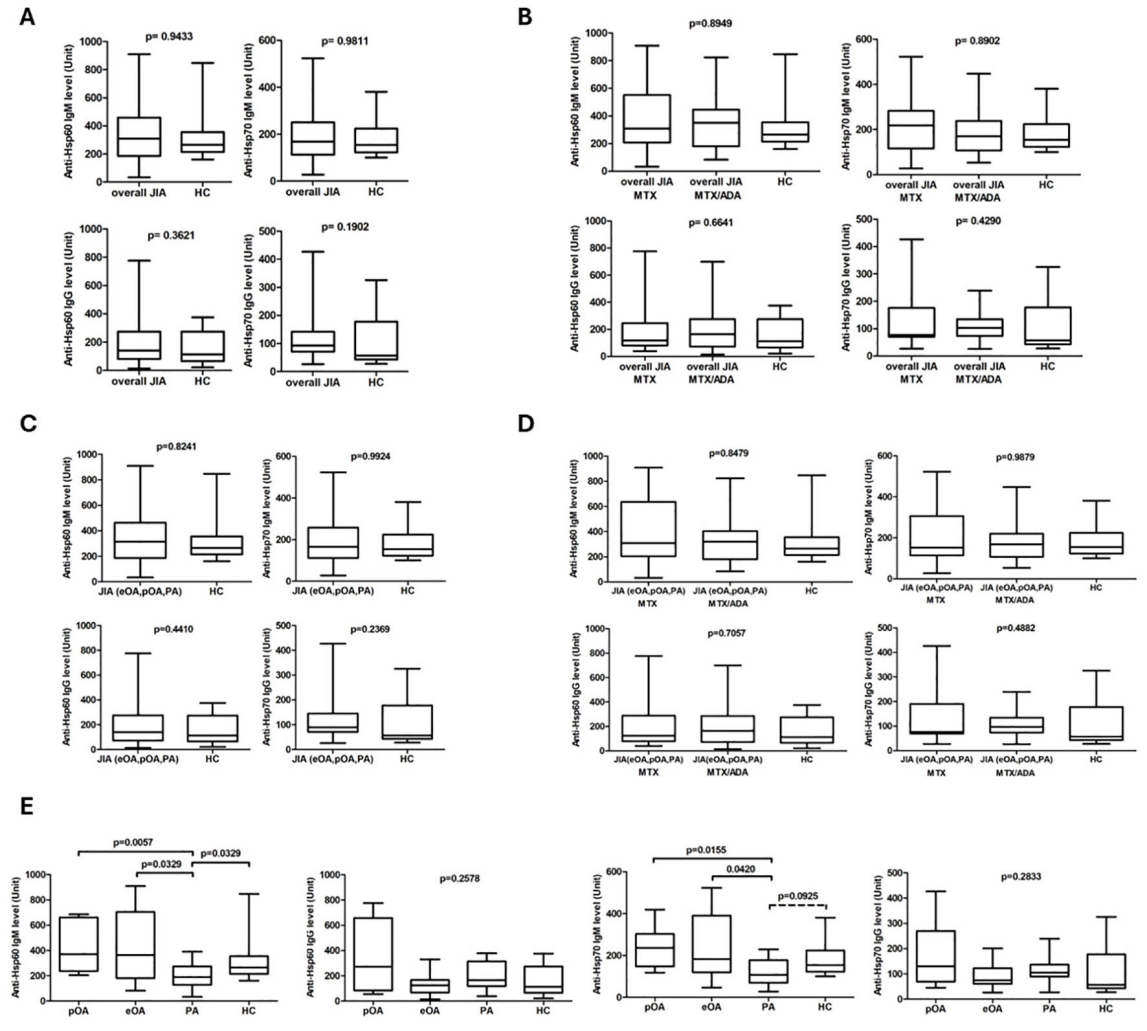
Natural autoantibody (nAAb) levels in juvenile idiopathic arthritis (JIA) and apparently healthy controls (HC). **(A)** Overall comparison between JIA (n=47) and HC (n=22). **(B)** Treatment-based analysis of overall JIA group MTX (n=19), MTX/ADA (n=28) and HC (n=22). **(C)** Comparison of between JIA groups (pOA: n=10; eOA: n=17; PA: n=10) and HC (n=22). **(D)** Treatment-based analysis of JIA groups (pOA, eOA, PA) MTX (n=15), MTX/ADA (n=22) and HC (n=22). **(E)** Severity-based analysis (eOA: n=17; pOA: n=10; PA: n=10; HC: n=22). eOA, extended oligoarticular JIA; ERA, Enthesitis-associated arthritis; HC, healthy control; JIA, juvenile idiopathic arthritis; MTX, methotrexate; MTX/ADA, methotrexate + adalimumab; n, number; PA, Polyarticular JIA; pOA, persistent oligoarticular JIA.

In the initial analysis, the entire juvenile idiopathic arthritis (JIA) cohort was compared with the apparently healthy control (HC) group. The JIA population included oligoarticular (eOA and pOA), polyarticular (PA), enthesitis-related (ERA), and systemic (sJIA) subtypes. No significant differences were observed in anti-Hsp60 or anti-Hsp70 IgM and IgG antibody levels between the overall JIA group and the apparently healthy controls (**Figure 2A**).

Patients were grouped according to treatment, and those receiving methotrexate alone and those receiving combined biological therapy with methotrexate were compared with the control group. Antibody levels against Hsp60 and Hsp70 (both IgM and IgG) showed similar distributions across all groups, and no statistically significant differences were detected (**Figure 2B**).

A further comparison focused on the most common JIA subtypes, based on frequency. The eOA, pOA, and PA subtypes were combined and compared with the control group; anti-Hsp60 and anti-Hsp70 IgM and IgG levels remained similar between the groups, with no significant differences (**Figure 2C**).

When the combined JIA group (eOA, pOA, and PA) was divided according to treatment (methotrexate alone versus combination therapy) and compared with the control group, antibody levels again showed comparable distributions, and no significant differences were observed (**Figure 2D**).

Disease severity was assessed by separately analyzing the eOA, pOA, and PA subgroups. The PA group showed significantly lower anti-Hsp60 IgM levels than the pOA (p = 0.0057) and eOA (p = 0.0329) groups. Similarly, anti-Hsp70 IgM levels were lower in the PA group than in the pOA and eOA groups (p = 0.0155 and p = 0.042, respectively). Compared with the HC group, the PA group showed lower anti-Hsp60 IgM levels (p = 0.0329) and a decreasing trend in anti-Hsp70 IgM levels (p = 0.0925) (**Figure 2E**). No significant differences were observed in IgG antibody levels in any subgroup comparison (**Figure 2**).

## 4 Discussion

Heat shock proteins are highly conserved chaperone molecules that are upregulated under cellular stress conditions and may be released during inflammation. In addition to their cytoprotective roles, Hsps are increasingly recognized as immunomodulatory molecules capable of interacting with both innate and adaptive immune responses (22, 23).

Previous studies have demonstrated the presence of anti-Hsp antibodies in autoimmune diseases (8, 10, 12, 16–19, 24–27). In JIA, Zlacka et al. reported circulating antibodies against Hsp60, Hsp65 and Hsp70 in patients, particularly in rheumatoid factor-positive patients, suggesting a relationship between anti-Hsp immune responses and disease severity (8). These findings support the concept that immune recognition of Hsps is not disease-specific but rather part of a broader immunological network associated with chronic inflammation and immune dysregulation.

In agreement with previous observations, no significant differences were observed between the overall JIA cohort and apparently healthy controls, suggesting that JIA, as a heterogeneous disease, is not associated with uniform alterations in nAAb levels across the entire population.

It is important to emphasize that our study did not aim to re-evaluate the presence of anti-Hsp reactivity in JIA, but rather to investigate whether different therapeutic approaches influence anti-Hsp natural autoantibody profiles in patients in remission.

We found comparable anti-Hsp60 and anti-Hsp70 antibody levels across treatment groups. This may be explained by the fact that both treatment strategies effectively controlled inflammation and maintained remission, thereby minimizing differences in systemic immune parameters. Furthermore, the immunomodulatory effects of methotrexate and TNF-α inhibition on B-cell function and the antibody repertoire may further contribute to the similarities observed between the treatment groups.

Both MTX and TNF-α inhibitors exert a broad range of immunomodulatory effects on innate and adaptive immune responses, including modulation of cytokine production, T-cell activation, and B-cell function (28, 29). These mechanisms may contribute to the comparable anti-Hsp antibody profiles observed across treatment groups. Previous studies have suggested that anti-inflammatory therapies may indirectly influence Hsp expression and Hsp-directed immune responses through suppression of chronic inflammatory signaling pathways (30, 31).

Schett et al. demonstrated altered Hsp70 expression and heat shock factor activation in rheumatoid arthritis synovial tissue, and that these changes were regulated by inflammatory cytokines and anti-inflammatory therapies (32). Furthermore, extracellular Hsp70 has been shown to inhibit TNF-α-induced proinflammatory mediator production in fibroblast-like synoviocytes (30).

These findings suggest that Hsp-specific immune responses may be influenced by inflammatory activity and its therapeutic modulation.

Importantly, in the context of autoimmune diseases, Hsp-specific immune responses are increasingly viewed as potentially protective rather than exclusively pathogenic. Hsp-derived peptides can induce regulatory T- cell (Treg) responses capable of suppressing chronic inflammation and promoting immunological tolerance (23, 33). Therefore, modulation of Hsp expression and anti-Hsp immunity during MTX or TNF-α inhibitor therapy may not only contribute to reducing inflammation but also to the broader restoration of immunological homeostasis.

Another observation of our study was that the observed differences were primarily associated with disease severity rather than the type of treatment. We compared the oligoarticular forms—in which fewer joints are affected and the course of the disease is milder (pOA and eOA)—with the more severe forms (PA) and the apparently healthy control group. While no significant changes were observed in Hsp60 and Hsp70 IgG antibodies in either comparison, significant alterations were observed in IgM antibodies between the groups. Both anti-Hsp60 and anti-Hsp70 IgM levels were significantly lower in the PA group than in the combined oligoarticular group. In addition, anti-Hsp60 IgM levels were significantly lower in the PA group than in the apparently healthy control group, while anti-Hsp70 IgM levels showed a similar decreasing trend in the PA group compared with the apparently healthy controls without reaching statistical significance (p=0.0925). These results suggest that alterations in natural IgM autoantibody levels may be more closely associated with disease severity and immune dysregulation than with the treatment regimen itself.

Therefore, reduced levels of anti-Hsp IgM autoantibodies in severe JIA may reflect an impaired immunoregulatory capacity and diminished control of chronic inflammation. Experimental evidence supports this concept, as the depletion of B1-cell derived IgM exacerbates inflammatory arthritis, whereas the restoration of natural IgM attenuates disease severity in animal models (34).

The potential protective role of natural IgM antibodies has also raised therapeutic interest. Although intravenous immunoglobulin (IVIG) therapy, predominantly based on IgG, is routinely used for autoimmune diseases, the potential application of IgM-containing immunoglobulins has also emerged. Although human data are currently limited, promising results have been obtained using experimental models. In a type 1 diabetes mouse model, the administration of polyclonal IgM from healthy donors reversed diabetes in a significant proportion of mice without inducing generalized immunosuppression (35).

## 5 Limitations and strengths

This study has several limitations, including its retrospective, single-center design, relatively small sample size, and subgroup imbalance. In addition, the cross-sectional assessment performed during remission does not allow for conclusions regarding longitudinal changes in anti-Hsp antibody levels during active disease or treatment response.

However, an important strength of this study is that only patients in clinical remission were included and categorized into well-defined treatment groups, thereby minimizing the confounding effects of active systemic inflammation.

Therefore, these findings should be considered hypothesis-generating and warrant further investigation of natural autoantibodies as potential biomarkers of disease severity in JIA.

## 6 Conclusion

In conclusion, our results suggest that reduced levels of natural IgM autoantibodies directed against Hsp60 and Hsp70 are associated with more severe JIA phenotypes during remission, whereas no overall differences were observed between the treatment groups or compared with apparently healthy controls. These findings support the potential role of natural IgM autoantibodies as markers of disease severity and immune dysregulation in JIA.

## Data Availability

The raw data supporting the conclusions of this article will be made available by the authors, without undue reservation.
